# Whole Genome Sequencing Reveals a Chromosome 9p Deletion Causing DOCK8 Deficiency in an Adult Diagnosed with Hyper IgE Syndrome Who Developed Progressive Multifocal Leukoencephalopathy

**DOI:** 10.1007/s10875-014-0114-4

**Published:** 2014-11-12

**Authors:** Aaron G. Day-Williams, Chao Sun, Ilijas Jelcic, Helen McLaughlin, Tim Harris, Roland Martin, John P. Carulli

**Affiliations:** 1 Translational Sciences, Biogen Idec, Cambridge, MA USA; 2 Department of Neurology, University Hospital Zurich, Zurich, Switzerland

**Keywords:** Hyper IgE Syndrome, DOCK8 deficiency, primary immune deficiency, Progressive Multifocal Leukoencephalopathy (PML), JC virus

## Abstract

**Purpose:**

A 30 year-old man with a history of recurrent skin infections as well as elevated serum IgE and eosinophils developed neurological symptoms and had T2-hyperintense lesions observed in cerebral MRI. The immune symptoms were attributed to Hyper IgE syndrome (HIES) and the neurological symptoms with presence of JC virus in cerebrospinal fluid were diagnosed as Progressive Multifocal Leukoencephalopathy (PML). The patient was negative for STAT3 mutations. To determine if other mutations explain HIES and/or PML in this subject, his DNA was analyzed by whole genome sequencing.

**Methods:**

Whole genome sequencing was completed to 30X coverage, and whole genome SNP typing was used to complement these data. The methods revealed single nucleotide variants, structural variants, and copy number variants across the genome. Genome-wide data were analyzed for homozygous or compound heterozygous null mutations for all protein coding genes. Mutations were confirmed by PCR and/or Sanger sequencing.

**Results:**

Whole genome analysis revealed deletions near the telomere of both copies of chromosome 9p. Several genes, including DOCK8, were impacted by the deletions but it was unclear whether each chromosome had identical or distinct deletions. PCR across the impacted region combined with Sanger sequencing of selected fragments confirmed a homozygous deletion from position 10,211 to 586,751.

**Conclusion:**

While several genes are impacted by the deletion, DOCK8 deficiency is the most probable cause of HIES in this patient. DOCK8 deficiency may have also predisposed the patient to develop PML.

**Electronic supplementary material:**

The online version of this article (doi:10.1007/s10875-014-0114-4) contains supplementary material, which is available to authorized users.

The hyper IgE syndromes (HIES) are rare primary immunodeficiencies characterized by elevated serum IgE, dermatitis and recurrent skin and lung infections [1, 2]. There are two forms of HIES that are characterized based on their inheritance patterns: autosomal-dominant HIES (AD-HIES) and autosomal-recessive HIES (AR-HIES). AD-HIES is caused by dominant mutations in STAT3 and is characterized in addition to the symptoms noted above by extra-immune manifestations including skeletal, connective tissue and vasculature abnormalities [1, 2]. AR-HIES is caused by homozygous or compound heterozygous mutations in DOCK8, TYK2 or STK3, and these patients do not possess any of the extra-immune manifestations found in AD-HIES [3–6]. The major manifestations of DOCK8 deficiency leading to AR-HIES are recurrent viral and bacterial infections starting early in life, extreme eosinophilia, and elevated IgE levels. The causes of DOCK8 deficiency thus far described range from point mutations and small indels to large deletions of portions of DOCK8 preventing expression of the protein [3–6]. This report adds to the growing body of knowledge about DOCK8-deficient AR-HIES and discovers the largest published deletion in the region around DOCK8. In addition, we add to the literature showing the occurence of Progressive Multifocal Leukoencephalopathy in DOCK8 deficient individuals.

A 30 year old, male, Caucasian patient suffering from eczematoid dermatitis developed impetiginization of the skin with Klebsiella pneumonia and group A β-hemolytic streptococcus species in July, 2008. The patient was diagnosed with HIES based on elevated serum IgE (26,800 kU/L) and elevated eosinophils (1.6 × 10^9^ /L). The patient had atopic dermatitis that was treated with topical corticosteroids and recurrent herpesviral skin infections from the age of 6 years. There were no reports of any other immunosuppressive agents. The patient history revealed that his parents were first cousins, however, no immunological diseases, opportunistic infection or childhood diseases were reported in the parent’s siblings or their children. In September, 2008 the patient developed a left-sided sensory hemisyndrome, which progressed to a spastic-atactic hemiparesis within a few weeks. A cerebral MRI showed a large confluent T2-hyperintense lesion in the frontal parietal central region of the right cerebral hemisphere, a small T2-hyperintense lesion in the right temporal cortex, and small T2-hyperintense lesions in the right cerebellar heimisphere. In October, 2008 the cerebrospinal fluid (CSF) of the patient was positive for JC polyoma virus DNA (500 copies/mL) leading to the diagnosis of Progressive Multifocal Leukoencephalopathy (PML). Peripheral blood analysis showed repeatedly highly increased numbers of IgE (12,166–26,800 kU/l) and eosinophils (3,066–6,068/ul) and decreased levels of lymphocytes (296–770/ul), CD3+ T cells (130–265/ul), CD4+ T cells (71–169/ul), CD8+ T cells (29–109/ul), CD19+ B cells (173–262/ul) and NK cells (10–81/ul) (Table S1). After stimulation of lymphocytes with phytohemagglutinin, CD3+ T cells responded adequately as shown by intracellular production of interferon-γ (24 % of cells), interleukin-2 (25.6 %) and tumor necrosis factor-α (11.2 %).

A screen for STAT3 mutations in April, 2009 was negative. The family history of first cousin parents and the absence of STAT3 mutations lead to the refined diagnosis of AR-HIES of unknown etiology. In August 2009, antiepileptic treatment with 3,000 mg levetiracetam and 100 mg pregabaline daily was started because of focal sensory epileptic seizures in the left hemibody. MRI follow-up showed a reduced size of the T2-lesion within the right cerebral central region, but CSF JCV DNA copy number had increased tenfold (5,200 JCV genomic copies/mL). Until December 2010, neurological deficits had progressed only mildly, but MRI showed dissemination and enlargement of the PML lesions in the left thalamic region, left hemisphere, pons, and the right cerebellar pedunculus. The patient’s DNA was sent for whole-genome SNP analysis and sequencing in April, 2012. After this the patient was not available for follow-up of the disease course.

The patient’s DNA was whole genome sequenced (WGS) by Complete Genomics Incorporated (CGI; software version 2.0) [7, 8] and was analyzed on the Illumina Omni 1 quad genome-wide SNP array. The WGS approach used short (31–35 base) sequence reads at >30X coverage mapped to the reference genome using methods previously described [7] to identify single nucleotide, copy number, and structural variants. Relative to the reference genome, the sequence of this individual included 19435 missense variants, 178 nonsense variants, 470 frameshift variants, and >100 copy number and structural variants. Single nucleotide variants (SNVs) were analyzed using the ENSEMBL Variant Effect Predictor v2.8 [9] on the ENSEMBL v70 database, and variant effects on the annotated canonical transcripts for all genes were assessed via PolyPhen2 [10] and SIFT [11]. A variant was considered possibly damaging if it was determined to be either ‘probably damaging’ or ‘possibly damaging’ by PolyPhen2 or ‘deleterious’ by SIFT. We examined known genes associated with immune deficiency, including the HIES genes STAT3, TYK2, STK3 and DOCK8, and observed no damaging mutations in STAT3 or STK3, heterozygosity for a possibly damaging missense variant in TYK2 (rs147991080, R448W, MAF <0.01 (http://browser.1000genomes.org/); Table S2), and large deletions on both copies of chromosome 9p in the region that includes DOCK8. The SNP array data also suggested a large deletion on chromosome 9p, with mostly non-called SNPs from approximately 194,000 to 600,000 bases (Table S3). Given the heterozygosity and ambiguous function of the TYK2 variant and the obvious deletions around DOCK8, it is clear that the DOCK8 mutation(s) contribute to the patient’s disease.

The WGS and SNP data were not clear on the exact deletion breakpoints or whether the patient was homozygous for the same deletion or had inherited two different deletions. The WGS CNV analysis estimated that the patient was homozygous null for DOCK8 and some of KANK1, but could be hemizygous towards the telomere encompassing CBWD1, FOXD4, FAM138C, WASH1, and DDX11L5 (Fig. 1 panels A,B), and was consistent with the hypothesis that the patient had some DNA telomeric to DOCK8. The WGS analysis of the p-arm from the telomere to the DOCK8 locus is complicated by a segmental duplication that results in extremely high sequence similarity to Chromosome 2 [12, 13] and ambiguous sequence assembly. To resolve the breakpoints for the individual’s deletion(s) we designed targeted PCR around SNPs rs12353065, rs7853676 and rs11794423 which the WGS SNP analysis called high confidence SNPs, the WGS CNV analysis estimated to be hemizygous, and PCR primers unique to chromosome 9 could be designed (Fig. 1 panels B,C). Figure 1 panel C shows that the PCR with primers for the 3 SNPs (gels a, b and c) are all negative illustrating that the patient is in fact homozygous null from around the telomere into KANK1. To resolve the exact breakpoints we designed PCR primers close to the telomere and in KANK1 (PCR pair d Fig. 1 panel B,C). Upon the successful amplification of this fragment we cloned the DNA and Sanger sequenced the fragment. We performed a global gapped alignment with the Needleman-Wunsch algorithm of the resulting DNA fragment to chromosome 9 from bases 1-700,000 (NC_000009.11) and it revealed the alignment in Supplementary Figure 1. The alignment shows a massive homozygous deletion from position 10,211 to 586,751 that makes the patient homozygous null for the genes WASH1, FAM138C, FOXD4, CWBD1, C9orf66, DOCK8 and most of KANK1.

**Fig. 1:**
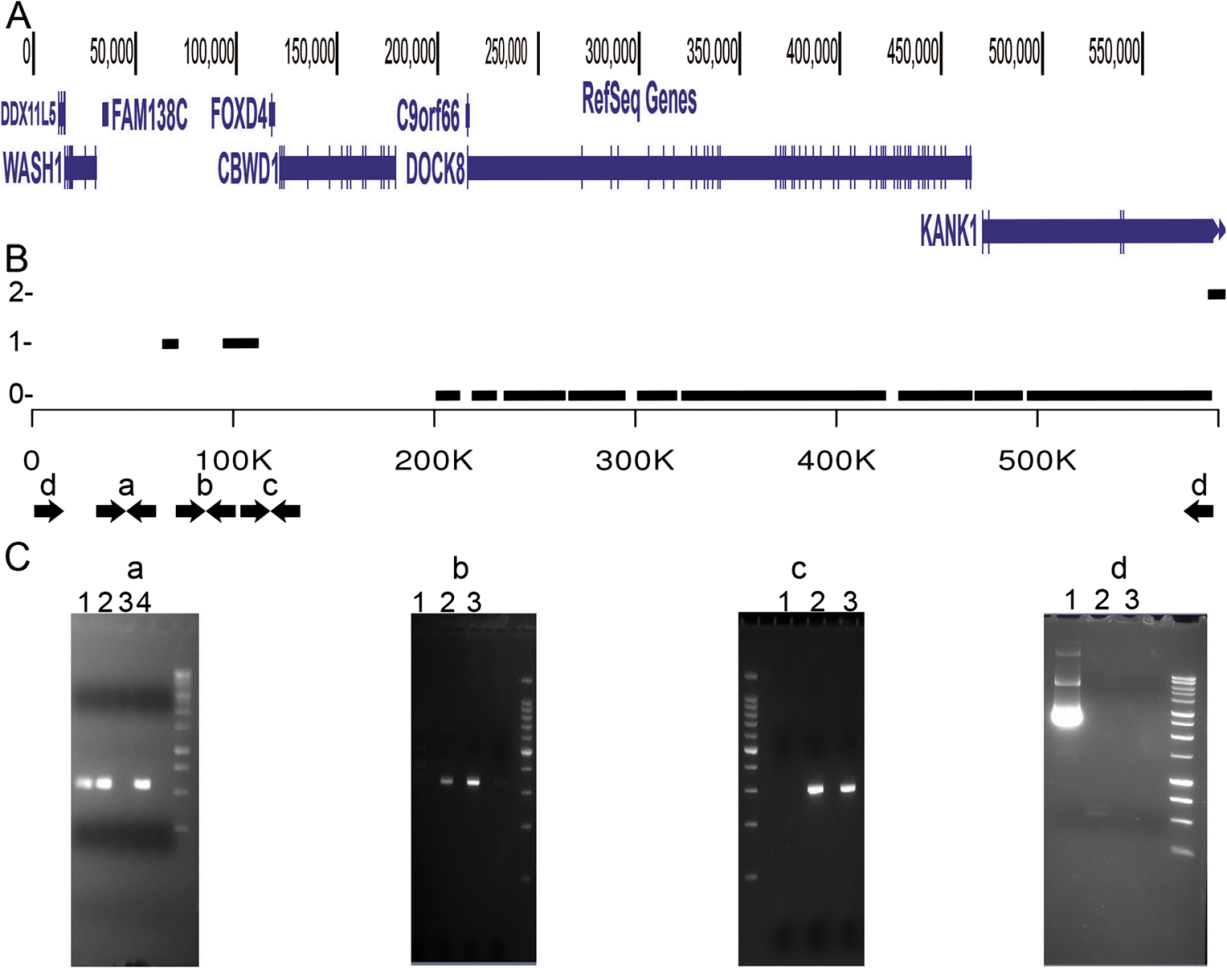
Deletion of DOCK8 in a patient with HIES and PML. *A*. Telomeric region of chromosome 9p showing the deleted region and the impacted genes. *B*. Hypothetical ploidy observed in whole genome sequence. Note apparent single copy coverage near the telomere, then nearly 500 kb with no coverage, and then diploid coverage starting at approximately 586,000 bases. The letters *a,b,c* and *d* show the approximate location of PCR primers used to confirm the nature of the deletion. *C*. Results of PCR using the primers shown in 1B. Gel a: lanes 1,2, and 4 are controls, lane 3 is the HIES subject. Gels b, c and d: lane 1 is the HIES subject, lanes 2 and 3 are control subject, lane 4 is a no template control. Primer sets a, b, and c produce bands from the controls but not from the HIES subject, and primer set d only produces a band from the HIES subject

DOCK8 deficiency is the most likely cause of HIES in this subject, and may have predisposed him to the development of PML. Among the genes in the deletion interval none of the other genes are so obviously connected to the phenotype (Table 1), and elsewhere in the genome there are no mutations consistent with known inheritance patterns for HIES. Notably, this is not the first case report of PML in DOCK8 deficiency [14]. PML has been observed in a limited subset of PIDs that includes DOCK8 deficiency, Wiskott-Aldrich Syndrome, STAT1 gain of function mutations and CD40L deficiency [15–20]. This observation highlights a new pathway by which the ubiquitous JC virus causes PML in a small fraction of individuals and further demonstrates the utility of whole genome sequencing for diagnosing diseases of unknown etiology. DOCK8 deficiency can be treated by bone marrow transplantation [21–23], and the possibility of PML in these individuals is a reason to consider early and accurate diagnosis of suspect cases by genetic analysis and treatment by transplantation.

**Table 1:** Genes in the deleted interval on chromosome 9, with Gene Ontology (GO;([24, 25]) biological process and function annotation, as well as associated phenotypes in Online Medelian Inheritance in Man (OMIM; http://omim.org/)

Gene Symbol	GO biological process	GO function	OMIM phenotypes (MIM number)
DDX11L5	None	None	None
WASH1	GO:0006810:transport GO:0016197:endosomal transport GO:0034314:Arp2/3 complex-mediated actin nucleation GO:0042147:retrograde transport, endosome to Golgi	GO:0003779:actin binding GO:0005515:protein binding GO:0031625:ubiquitin protein ligase binding GO:0043014:alpha-tubulin binding	None
FAM138C	None	None	None
FOXD4	GO:0006351:transcription, DNA-templated GO:0006355:regulation of transcription, DNA-templated	GO:0003677:DNA binding GO:0003700:sequence-specific DNA binding transcription factor activity GO:0008301:DNA binding, bending GO:0043565:sequence-specific DNA binding	None
CBWD1	None	GO:0000166:nucleotide binding GO:0005524:ATP binding	None
C9orf166	None	None	None
Dock8	GO:0001771;immunological synapse formation GO:0007264;small GTPase mediated signal transduction GO:0007596:blood coagulation GO:0036336:dendritic cell migration GO:0043547:positive regulation of GTPase activity GO:0061485:memory T cell proliferation GO:0070233:negative regulation of T cell apoptotic process	GO:0005085:guanyl-nucleotide exchange factor activity GO:0005515:protein binding	Hyperimmunoglobulin E recurrent infection syndrome, autosomal recessive (243700) Mental retardation, autosomal dominant 2 (614113)
KANK1	None	None	Cerebral palsy, spastic quadriplegic, 2 (612900)

## Electronic supplementary material

Below is the link to the electronic supplementary material.

ESM 1 (DOCX 17 kb)

ESM 2 (DOCX 14 kb)

ESM 3 (XLSX 11 kb)

ESM 4 (XLSX 23 kb)
