# Supplementary material for: Whole Genome Sequencing Reveals a Chromosome 9p Deletion Causing DOCK8 Deficiency in an Adult Diagnosed with Hyper IgE Syndrome Who Developed Progressive Multifocal Leukoencephalopathy
Source: J Clin Immunol. 2014 Nov 12;35(1):92–6. doi: 10.1007/s10875-014-0114-4 (PMC4306731; doi:10.1007/s10875-014-0114-4)
Supplement: Supplementary file 1 — (DOCX 17 kb) [file 10875_2014_114_MOESM1_ESM.docx]

A

00001 gtagtctggctctagggtgtgtctgcttaactacactattcccattgttttacctccctccctcccaccaacccatccattcacccacccattcatccaa 00100

>>>>> |||||||||||||||||||||||||||||||||||||||||||||||||||||||||||||||||||||||||||||||||||||||||||||||||||| >>>>>

48364 gtagtctggctctagggtgtgtctgcttaactacactattcccattgttttacctccctccctcccaccaacccatccattcacccacccattcatccaa 48463

00101 acatccacccatacatccatccatctgcccacccacctatgcatccatctcgagtttcatgtaagagtttatttaataaaggaattctgaagaatgataa 00200

>>>>> |||||||||||||||||||||||||||||||||||||||||||||||||||||||||||||||||||||||||||||||||||||||||||||||||||| >>>>>

48464 acatccacccatacatccatccatctgcccacccacctatgcatccatctcgagtttcatgtaagagtttatttaataaaggaattctgaagaatgataa 48563

00201 aaaataaaaacttgaaaactactcaagattgctatttaaactgcaaaagtcctcaggggtggggttctatgactcacattgcacttagaataaagagaaa 00300

>>>>> |||||||||||||||||||||||||||||||||||||||||||||||||||||||||||||||||||||||||||||||||||||||||||||||||||| >>>>>

48564 aaaataaaaacttgaaaactactcaagattgctatttaaactgcaaaagtcctcaggggtggggttctatgactcacattgcacttagaataaagagaaa 48663

00301 ttctttttatacaatataagttcctgcagaatgcagacactttctacttctccagcctcttttcgactcctctcctactggcttctgtatttaagccaca 00400

>>>>> |||||||||||||||||||||||||||||||||||||||||||||||||||||||||||||||||||||||||||||||||||||||||||||||||||| >>>>>

48664 ttctttttatacaatataagttcctgcagaatgcagacactttctacttctccagcctcttttcgactcctctcctactggcttctgtatttaagccaca 48763

00401 ttagacctttcttcagtttttatatcgactttgttgcatcacacctcagagattctgtacatgttcttcctcctgcctagaaagggtcgtccctccactt 00500

>>>>> |||||||||||||||||||||||||||||||||||||||||||||||||||||||||||||||||||||||||||||||||||||||||||||||||||| >>>>>

48764 ttagacctttcttcagtttttatatcgactttgttgcatcacacctcagagattctgtacatgttcttcctcctgcctagaaagggtcgtccctccactt 48863

00501 tcgccaactaatccctgctcaacttttcatctcagcagga 00540

>>>>> |||||||||||||||||||||||||||||||||||||||| >>>>>

48864 tcgccaactaatccctgctcaacttttcatctcagcagga 48903

B

00001 gtgcttcgttaaacagatgcttgaaggcagcatgctcgttaagagtcatcaccactccctaatctcaagtacccagggacacaaacactgcggaaggccg 00100

<<<<< |||||||||||||||||||||||||||||||||||||||||||||||||||||||||||||||||||||||||||||||||||||||||||||||||||| <<<<<

83143 gtgcttcgttaaacagatgcttgaaggcagcatgctcgttaagagtcatcaccactccctaatctcaagtacccagggacacaaacactgcggaaggccg 83044

00101 ctgggtcctctgcctaggaaaaccagagaccttggttcacttgtttatctgctgaccttccctccactattgtcctatgaccctgccaaatccccctctg 00200

<<<<< |||||||||||||||||||||||||||||||||||||||||||||||||||||||||||||||||||||||||||||||||||||||||||||||||||| <<<<<

83043 ctgggtcctctgcctaggaaaaccagagaccttggttcacttgtttatctgctgaccttccctccactattgtcctatgaccctgccaaatccccctctg 82944

00201 caagaaacacccaagaatgatcaataaaaaaaaaaaaaaaaaaaaaaaaaaa 00252

<<<<< ||||||||||||||||||||||||||||||||||||||||||||||||| || <<<<<

82943 caagaaacacccaagaatgatcaataaaaaaaaaaaaaaaaaaaaaaaagaa 82892

C

000002 tttgggatgttttcctctgcttgtttctacgcctttgcaacaacgtccggcaaagatgccttcgccttttataaaagcttcttcaagaccatgtgtggtg 000101

>>>>>> |||||||||||||||||||||||||||||||||||||||||||||||||||||||||||||||||||||||||||||||||||||||||||||||||||| >>>>>>

118236 tttgggatgttttcctctgcttgtttctacgcctttgcaacaacgtccggcaaagatgccttcgccttttataaaagcttcttcaagaccatgtgtggtg 118335

000102 gactcccccctttataacccttcttcccctacctcggagcggtgccacttcctcctaacgtagtccagggatgatggtcttctgggcaaacaccgtccgg 000201

>>>>>> ||| || ||||||||||||||||||||||||||||||||||||||||||||||||||||||||||||||||||||||||||||||||||||||||||||| >>>>>>

118336 gacgcctccctttataacccttcttcccctacctcggagcggtgccacttcctcctaacgtagtccagggatgatggtcttctgggcaaacaccgtccgg 118435

000202 agaaaagcccagccccctcctcctcgcacccacctgccaccaaggaagatgctctactcatccggtgcagccagacagtaggcaagcctttgca 000295

>>>>>> ||||||||||||| |||||||||||||||||||||||||||||||||||||||||||||||||||||||||||||||||||||||||||||||| >>>>>>

118436 agaaaagcccagcgccctcctcctcgcacccacctgccaccaaggaagatgctctactcatccggtgcagccagacagtaggcaagcctttgca 118529

D

1 G-----------------------------------AATTCGCCCTTCAACCCAACCCTAACCCTAACCCTAACCC-------------------------------------------- 41

>>>>> || | | |||||||||||||||||||||||||||||

10000 NTAACCCTAACCCTAACCCTAACCCAACCCCACCCCAACCCCAACCCCAACCCAACCCTAACCCTAACCCTAACCCAACCCTAACCCTAACCCTAACCCAACCCTCACCCTCACCCTCAC 10119

42 ------------------------------------------------------------------------------TAACCCTAACCATAC--------------------------- 56

>>>>> ||||||||||| |||

10120 CCTCACCCTCACCCTCACCCTCACCCTAACCCTACCCTAACCCCTAACCCCTAACCCCTAACCCCTAACCCTTAACCCTAACCCTAACCCTACCCTAACCCTAACCCTAACCCCTAACCC 10239

57 -------------------------------GCTTTTCTTTGCAGTGGTGGAGAGTCACGAGAGCAAATAGCTAGCCTTTACCTAAAGCAAAAGGGACTCTTTGCATGATATCTTAGGCT 145

>>>>>> |||||||||||||||||||||||||||||||||||||||||||||||||||||||||||||||||||||||||||||||||||||||||

586720 ACAGAAGGTGGAAAGCTATGAGGGATCCAGCGCTTTTCTTTGCAGTGGTGGAGAGTCACGAGAGCAAATAGCTAGCCTTTACCTAAAGCAAAAGGGACTCTTTGCATGATATCTTAGGCT 586839

146 CCCTTTTATTCTGGCCTGCCCCTGCACCCCCAGCAGGAAAGTGGCTTAACTGTACTTTGATTTCCAAAATGCGTAGCAGTCTCTAGTATTGATTCCCCTCTCCTGCAGATCTCAAGCCTA 265

>>>>>> ||||||||||||||||||||||||||||||||||||||||||||||||||||||||||||||||||||||||||||||||||||||||||||||||||||||||||||||||||||||||

586840 CCCTTTTATTCTGGCCTGCCCCTGCACCCCCAGCAGGAAAGTGGCTTAACTGTACTTTGATTTCCAAAATGCGTAGCAGTCTCTAGTATTGATTCCCCTCTCCTGCAGATCTCAAGCCTA 586959

...

**Supplementary Figure 1**. Sequence Alignments to Chromosome 9 for PCR Fragments from Figure 1. The top lines are the sequences from the gel and the bottom lines are sequences from the chromosome 9 reference genome on build37 of the genome. **A**. BLAT alignment of the PCR fragment from control individual in lane 1 of gel a in Figure 1C. **B**. BLAT alignment of the PCR fragment from control individual in lane 2 of gel b in Figure 1C. **C**. BLAT alignment of the PCR fragment from control individual in lane 2 of gel c in Figure 1C. **D**. Sequence of the junction fragment spanning the Chromosome 9p deletion in the HIES patient. Needleman-Wunsch alignment of the PCR fragment from HIES patient in lane 1 of gel d in Figure 1C. The deletion is from position 10,211 to 586,751. The alignment extends to position 589271 of chromosome 9.
