# Supplementary material for: Whole Genome Sequencing Reveals a Chromosome 9p Deletion Causing DOCK8 Deficiency in an Adult Diagnosed with Hyper IgE Syndrome Who Developed Progressive Multifocal Leukoencephalopathy
Source: J Clin Immunol. 2014 Nov 12;35(1):92–6. doi: 10.1007/s10875-014-0114-4 (PMC4306731; doi:10.1007/s10875-014-0114-4)
Supplement: Supplementary file 2 — (DOCX 14 kb) [file 10875_2014_114_MOESM2_ESM.docx]

Supplementary Table 1. Serum IgE and blood cell counts.

Serum IgE:

22.07.2008 = 26,800 kU/l

06.05.2009 = 14,384 kU/l

25.06.2009 = 12,166 kU/l

12.07.2009 = 13,274 kU/l

13.08.2009 = 18,011 kU/l

14.09.2009 = 14,209 kU/l

09.10.2009 = 15,839 kU/l

18.01.2011 = 21,273 kU/l

Eosinophiles:

22.07.2008 = 1600/ul (norm. >450/ul)

20.11.2008 = 3066/ul (norm. 50-360/ul)

05.03.2009 = 3882/ul (norm. 50-360/ul)

03.04.2009 = 5270/ul (norm. 50-360/ul)

06.05.2009 = 4521/ul (norm. 50-360/ul)

25.06.2009 = 4446/ul (norm. 50-360/ul)

03.07.2009 = 2038/ul (norm. 50-360/ul)

12.07.2009 = 5183/ul (norm. 50-360/ul)

17.07.2008 = 3109/ul (norm. 50-360/ul)

13.08.2009 = 6068/ul (norm. 50-360/ul)

09.10.2009 = 7860/ul (norm. 50-360/ul)

Lymphocytes:

22.07.2008 = 700/ul (norm. 1100 – 4800/ul)

20.11.2008 = 365/ul (norm. 1320 – 3570/ul)

05.03.2009 = 333/ul (norm. 1320 – 3570/ul)

03.04.2009 = 620/ul (norm. 1320 – 3570/ul)

06.05.2009 = 733/ul (norm. 1320 – 3570/ul)

25.06.2009 = 585/ul (norm. 1320 – 3570/ul)

03.07.2009 = 326/ul (norm. 1320 – 3570/ul)

12.07.2009 = 682/ul (norm. 1320 – 3570/ul)

17.07.2008 = 322/ul (norm. 1320 – 3570/ul)

13.08.2009 = 296/ul (norm. 1320 – 3570/ul)

09.10.2009 = 770/ul (norm. 1320 – 3570/ul)

CD3 T cells:

03.04.2009 = 265/ul (norm. 590-1990/ul)

06.05.2009 = 248/ul (norm. 590-1990/ul)

12.07.2009 = 107/ul (norm. 590-1990/ul)

13.08.2009 = 130/ul (norm. 590-1990/ul)

CD4 T cells:

03.04.2009 = 169/ul (norm. 440-1100/ul)

06.05.2009 = 148/ul (norm. 440-1100/ul)

12.07.2009 = 73/ul (norm. 440-1100/ul)

13.08.2009 = 71/ul (norm. 440-1100/ul)

CD8 T cells:

03.04.2009 = 104/ul (norm. 210-750/ul)

06.05.2009 = 109/ul (norm. 210-750/ul)

12.07.2009 = 29/ul (norm. 210-750/ul)

13.08.2009 = 59/ul (norm. 210-750/ul)

activated T cells

12.07.2009 = 46/ul (norm. 50-270/ul)

NK cells

03.04.2009 = 81/ul (norm. 80-350/ul)

12.07.2009 = 23/ul (norm. 80-350/ul)

13.08.2009 = 10/ul (norm. 80-350/ul)

CD19 B cells:

03.04.2009 = 262/ul (norm. 110-530/ul)

12.07.2009 = 173/ul (norm. 110-530/ul)

13.08.2009 = 166/ul (norm. 110-530/ul)

31.03.2009: After stimulation of lymphocytes with the mitogen phytohemagglutinin, CD3+ T cells responded adequately as shown by intracellular production of interferon-γ (24% of cells), interleukin-2 (25.6%) and tumor necrosis factor-α (11.2%).
